# Supplementary material for: Improved Adipose Tissue Function after Single Anastomosis Duodeno-Ileal Bypass with Sleeve-Gastrectomy (SADI-S) in Diet-Induced Obesity
Source: Int J Mol Sci. 2022 Oct 1;23(19):11641. doi: 10.3390/ijms231911641 (PMC9570280; doi:10.3390/ijms231911641)
Supplement: Supplementary file 1 [file ijms-23-11641-s001.zip › ijms-1861338-supplementary.pdf]

Supplemental Table S1. Body weight, adiposity and metabolic profile in control groups.

Determination	Normal diet (n=10)	High-fat diet (n=10)	*P*
Body weight (g)	504 ± 10	536 ± 16	**0.021**
Epididymal WAT (g/100 g BW)	1.19 ± .07	1.79 ± 0.14	**0.041**
Subcutaneous WAT(g/100 g BW)	1.00 ± 0.07	1.35 ± 0.14	**0.048**
Perirrenal WAT (g/100 g BW)	1.17 ± 0.05	2.08 ± 0.16	**0.025**
BAT (g/100 g BW)	0.127 ± 0.009	0.156 ± 0.017	0.150
Total WAT (g/100 g BW)	3.36 ± 0.15	5.22 ± 0.41	**0.004**
Rectal temperature (ºC)	36.1 ± 0.1	37.1 ± 0.0	**0.003**
Glucose (mg/dL)	63 ± 5	75 ± 4	**<0.001**
Insulin (ng/mL)	5.0 ± 0.6	8.0 ± 1.2	**0.046**
HOMA	1.0 ± 0.2	1.6 ± 0.3	**0.040**
QUICKI	0.39 ± 0.01	0.36 ± 0.01	**0.050**
FFA (mg/dL)	18 ± 2	20 ± 2	0.294
TG (mg/dL)	143 ± 15	142 ± 14	0.934
Cholesterol (mg/dL)	55 ± 3	70 ± 5	0.092
Glycerol (mg/dL)	0.023 ± 0.001	0.026 ± 0.002	0.225
Adipo-IR index	25.6 ± 0.3	47.4 ± 0.6	**0.035**
Leptin (ng/mL)	5.1 ± 0.7	10.2 ± 1.5	**0.032**
Adiponectin (ng/mL)	1.8 ± 0.3	0.8 ± 0.4	**0.050**
Adpn/leptin ratio	0.42 ± 0.09	0.14 ± 0.08	**0.042**

BW, body weight; WAT, white adipose tissue; Adpn, adiponectin; FFA, free fatty acids; HOMA, homeostasis model assessment; IPITT, intraperitoneal insulin tolerance test; QUICKI, quantitative insulin sensitivity check index; TG, triglycerides. Values presented as the mean ± S.E.M. Statistical differences were analyzed by the Student's *t* test.

Supplemental Table S2. Sequences of the primers and TaqMan® probes.

Gene (GenBank accession number)	Oligonucleotide sequence (5'-3')
Ask1 (NM_001277694)	
Forward	GCAACCAGGTCAGAATTGCTATTAA
Reverse	CTTGTGCTTCAGATGCTTGTGTAAG
TaqMan® Probe	FAM-ACAGCAGATACTCTCAGCCCCTGCATGA-TAMRA
Atf7 (NM_001108115.1)	
Forward	TGGGCCAGGTATACCAGGAAT
Reverse	GCCAGGGCCTCCAGTACAG
TaqMan® Probe	FAM-TATTCGCTGGCCAGA-TAMRA
Bmp7 (NM_001191856)	
Forward	CCTGCAAGAAACACGAGCCTGT
Reverse	AGGCACACTCTCCCTCACAGT
TaqMan® Probe	FAM-CTGGCAGGACTGGATCA-TAMRA
Cd137 (NM_001025773)	
Forward	CTGTGGCTTGGGAACGTTTAAT
Reverse	TCTTTCTCCTTGGTCCCATTCTT
TaqMan® Probe	FAM-CCGACCCTGGACGAACTGCTCTCTAGA-TAMRA
Dio2 (NM_031720.5)	
Forward	ACGCCTACAAACAGGTTAAATTG
Reverse	AATTGTTACCTGCTTCAGGATTG
TaqMan® Probe	FAM-CTCCCAATTCCAGTGTG-TAMRA
Fgf21 (NM_130752.1)	
Forward	CTCTATGGATCGCCTCACTTTGA
Reverse	GCTGGATCCTGGGAGTCCTT
TaqMan® Probe	FAM-CCTGAGGCCTGCAGTTTCAGAGAGCTG-TAMRA
Pgc1a (NM_031347)	
Forward	CGAACCTTAAGTGTGGAACTC
Reverse	GGGTTATCTTGGTTGGCTTTA
TaqMan® Probe	FAM-AACTGCAGGCCTAATC-TAMRA
Pparg (NM_013124)	
Forward	CTGACCCAATGGTTGCTGATTAC
Reverse	CCTGTTGTAGAGTTGGGTTTTTTCA
TaqMan® Probe	FAM-CCTGAAGCTCCAAGAATACCAAAGTGCGA-TAMRA
Prdm16 (NC_005104)	
Forward	AGTGGCAAGCGCTTCGAA
Reverse	ATGTGACGCTGGAGGTTGCT
TaqMan® Probe	FAM-AAACTGCGTCAAGGTGTT-TAMRA
Pref1 (NM_053744.2)	
Forward	GATATTCGGGGCTTGCACCTCTA
Reverse	GGAGCATTCGTACTGGCCTTT
TaqMan® Probe	FAM-CCCTGCGCGCCAACAATG-TAMRA
Sirt1 (NM_001107627)	
Forward	CTGGAGCTGGGGTTTCTGTTT
Reverse	CGAACATGGCTTGAGGATCTG
TaqMan® Probe	FAM-TTATGCTCGCCTTGCTGTGGACTTCC-TAMRA
Sirt3 (NM_001106313)	
Forward	GACATACGGGCTGACGTGATG
Reverse	GGCGAAATCAGCCACATGTAGT
TaqMan® Probe	FAM-TGCCCCGCTGCCCTGTCTGTACT-TAMRA
Sirt6 (NM_001031649.1)	
Forward	ATSCGCGGATAAGGGCAAGTG
Reverse	CACAGTGGAGGACTGCCACAT

TaqMan® Probe	FAM-TGCCGAGATATTCGAC-TAMRA
Slc27a1 (NM_053580)	
Forward	GGCCAACGATGTGCTCTATGA
Reverse	CACCGTTAACCCGTAGATGATACAC
TaqMan® Probe	FAM-CTCTACCACTCAGCAGGGAACATCATGGG-TAMRA
Tbx1 (NM_001108322)	
Forward	AGCTGGGCACCGAGATGAT
Reverse	GGTCCATTCCAAAAAGCTTCACT
TaqMan® Probe	FAM- CAGGCAGACGAATGTTCCCCACCTT -TAMRA
Tmem26 (NM_001107623)	
Forward	CCGAGGCTACAAATGGCTTTC
Reverse	ACTGGTTTCCATGGTGCATTTC
TaqMan® Probe	FAM- CCTGATCAACATCATGCCGTCCCTGT -TAMRA
Ucp1 (NM_012682)	
Forward	TCCCTCAGGATTGGCCTCTAC
Reverse	GCCAGCCGAGATCTTGCTT
TaqMan® Probe	FAM-TCTTCAGGGAGAAC -TAMRA
Ucp3 (NM_013167)	
Forward	GCAGTTCTACACCCCAAGG
Reverse	TGCAGCCTGCAGAATCC
TaqMan® Probe	FAM-ACGGACCACTCCAGCGT-TAMRA
Xbp1 (NC_005113)	
Forward	CTGACGCTGTTGCCTCTTCAG
Reverse	ACAGGGTCCAACTTGTCCAGAAT
TaqMan® Probe	FAM-TTCTGAGTCTGATATCCTTTTG-TAMRA

Ask1, apoptosis signal-regulating kinase 1; *Atf7*, activating transcription factor 7; *Bmp7*, bone morphogenetic protein 7; *Dio2*, iodothyronine deiodinase 2; *Fgf21*, fibroblast growth factor 21; *Pgc1a*, peroxisome proliferative activated receptor coactivator 1 a; *Pparg*, peroxisome proliferator-activated receptor gamma; *Prdm16*, PR domain containing 16; Pref, preadipocyte factor 1; *Sirt*, sirtuin; *Slc27a1*, solute carrier family 27 member 1; *Tbx1*, T-Box transcription factor 1; *Tmem26*; transmembrane protein 26; *Ucp*, uncoupling protein; *Xbp1*, X-box binding protein 1.

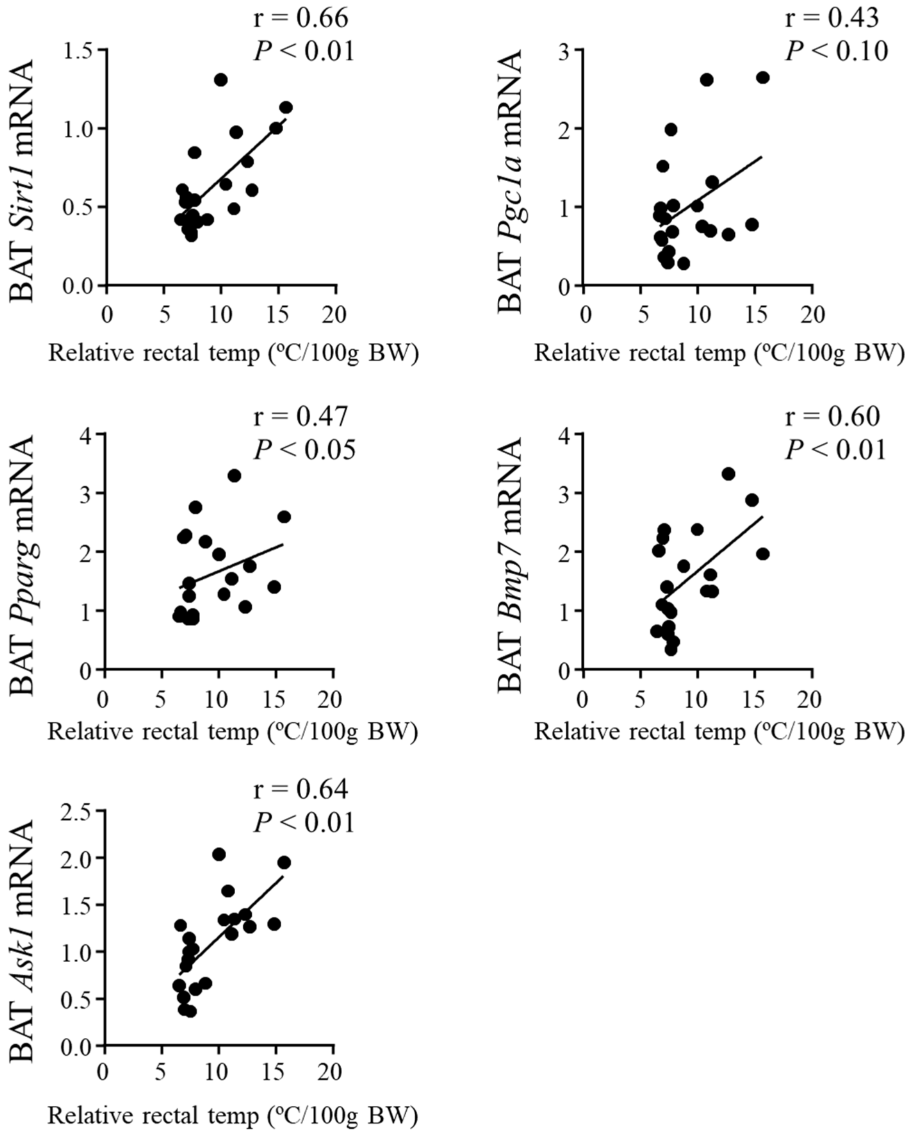

Supplemental Figure S1. Scatter diagrams showing the positive association between normalized rectal temperature and (a) *Sirt1*, (b) *Pgc1a*, (c) *Pparg*, (d) *Bmp7* and (e) *Ask1* mRNA levels. Pearson's correlation coefficient and *P* value are indicated. BW, body weight.
